# Acceptance and Feasibility of a Guideline for the Animal Welfare Assessment of Fattening Pigs from Farmers’ Point of View

**DOI:** 10.3390/ani10040711

**Published:** 2020-04-19

**Authors:** Mareike Pfeifer, Alexandra Koch, Clara Lensches, Armin O. Schmitt, Engel F. Hessel

**Affiliations:** 1 Department of Animal Sciences, Georg-August-University of Göttingen, Gutenbergstraße 33, D-37075 Göttingen, Germany; 2 Breeding Informatics, Georg-August-University of Göttingen, Margarethe von Wrangell-Weg 7, D-37075 Göttingen, Germany; armin.schmitt@uni-goettingen.de; 3 Center for Integrated Breeding Research (CiBreed), Georg-August-University of Göttingen, Albrecht-Thaer-Weg 3, D-37075 Göttingen, Germany; 4 Thünen-Institute of Agricultural Technology, Federal Research Institute for Rural Areas, Forestry and Fisheries, Bundesallee 47, D-38116 Braunschweig, Germany; engel.hessel@thuenen.de

**Keywords:** animal welfare evaluation, animal welfare indicators, feasibility, acceptance

## Abstract

**Simple Summary:**

The German Association for Technology and Structures in Agriculture has published the guide “Animal Welfare Indicators: Practical Guide—Pigs”, which is intended to help farmers to evaluate the welfare of pigs. Crucial for the acceptance of the guide by farmers is a high degree of feasibility of the contained indicators as well as the proposed procedure for recording them. To evaluate this, 40 farmers keeping fattening pigs were interviewed. The result is that, apart from faecal soiling and tail length, all the other eleven indicators are accepted for the assessment of fattening pig welfare by a majority of the interviewed farmers (between 57.5% and 90% acceptance per indicator). The feasibility of the individual indicators is overall assessed as positive. However, the study also shows a need for revision of the guide and makes suggestions for its improvement.

**Abstract:**

The welfare of farm animals is being increasingly discussed in society and politics. To evaluate animal welfare, indicator systems are often used. Such a system has been developed by the German Association for Technology and Structures in Agriculture and suggested in the publication “Animal Welfare Indicators: Practical Guide—Pigs”. The association’s aim is to provide farmers with a useful method for recording the welfare of pigs. Crucial for the acceptance of the guide by farmers is a high degree of feasibility of the recommended indicators as well as the proposed methods for their recording. To evaluate this, 40 farmers keeping fattening pigs were interviewed. The guided semi-structured interview was conducted on the farms after the farmers evaluated the welfare of their fattening pigs according to the guide. The results are: Apart from the indicators faecal soiling and tail length, all the other eleven indicators are accepted for the assessment of fattening pig welfare by a majority of the interviewed farmers (between 57.5% and 90% acceptance per indicator). Furthermore, the feasibility of the individual indicators was assessed as being positive. The relationship between time expenditure and benefit was rated on a five-point scale at an average of 3.1 (medium), which clearly shows that there is a need for further development of this guide. Some possible changes with a potential for improvement could be identified; for example, the aggregation of the results after the collection of the individual indicators to an overall result that can be compared and interpreted.

## 1. Introduction

The welfare of farm animals is often perceived as debateable by the general public [1] and has increasingly been the subject of social and political discourse in recent decades [2,3]. In many member states of the European Union, the legal framework for livestock farming has been changed in order to improve animal welfare [4]. In Germany, owners of livestock have been legally obliged to undertake an on-farm self-assessment regarding animal welfare since February 2014 [5]. In § 11 Art. 8 of the German Animal Welfare Act, the legislators demand the collection of suitable animal-based welfare indicators [6], thus emphasising the personal responsibility of the farmer for the animal’s welfare [7]. As the legislators have not made any specific stipulations with respect to the content, scope and frequency of the on-farm self-assessment, the German Association for Technology and Structures in Agriculture (KTBL, Kuratorium für Technik und Bauwesen in der Landwirtschaft e.V.) has published a proposal in 2016. This proposal contains both indicators for the animal welfare assessment of sows, suckling piglets, rearing piglets and fattening pigs as well as recommendations for their collection and was published as the KTBL publication “Animal Welfare Indicators: Practical Guide—Pigs” [8]. Although farmers can in principle delegate the on-farm self-assessment to third parties such as veterinarians or other specialists [5], the KTBL publication is primarily aimed directly at the farmers themselves. An important factor for the acceptance of the KTBL publication by farmers is a high degree of feasibility of both the animal welfare indicators it contains as well as the proposed methods for recording them. For this reason, the KTBL tried to define indicators that can be collected and documented easily and with as little effort as possible and to develop a proposal for the on-farm self-assessment that has a positive cost-benefit ratio for the farmers [5]. Whether the KTBL was able to achieve those targets is examined in the present study. The aim is to investigate the acceptance and feasibility of the KTBL proposal for the animal welfare assessment of fattening pigs from the perspective of livestock farmers. The investigation of further aspects that are mentioned in the scientific context for evaluating the feasibility of animal welfare assessment schemes, however, is beyond the scope of this study. These are for example: (1) required input from farmers should be as little as possible [9] in case of external control or delegation of on-farm self-assessment to a third party; (2) possibility of flexible application in different types of animal husbandry [10] and (3) a low degree of subjectivity, so that a valid result can be achieved by assessors who are not especially trained [10]. Regarding the degree of comparability after collection by different untrained assessors, some indicators that are mentioned in the KTBL guide for animal welfare assessment of fattening pigs have already been investigated [11].

## 2. Materials and Methods

### 2.1. The KTBL Guide “Animal Welfare Indicators: Practical Guide—Pigs”

The KTBL guide contains twelve animal-based and one resource-based indicator for the animal welfare assessment of fattening pigs. All indicators are listed in Table 1 in which the suggested time and frequency of their recording as well as information on which and how many fattening pigs are to be taken into account is included. The animal-based indicators can be divided into two categories: The indicators animal losses, antibiotic treatment index, daily weight gains and slaughter checks are supposed to be evaluated for the entire fattening pig herd. The other eight animal-based indicators are supposed to be collected for individual fattening pigs. The indicator tail length is assessed in all newly housed animals. In order to collect the indicators faecal soiling, runts, skin lesions, ear lesions, tail lesions, lameness, and evidence of ectoparasites a random sample of the fattening pigs shall be drawn and evaluated in herds with more than 150 pigs. In herds with less than 150 pigs, sampling is not recommended and these indicators are therefore collected for all individual fattening pigs in those herds. The KTBL guide gives the following instructions for the sampling: First of all, ten pens should be chosen, taking different age and weight classes of the pigs into account proportionately; the hospital pens should not be included. All fattening pigs in the selected pens should then be evaluated. However, in pens containing more than 15 pigs, the indicators skin lesions, ear lesions, tail lesions, and evidence of ectoparasites should only be recorded for 15 randomly selected pigs. If less than ten pens are available, the number of fattening pigs considered per pen should be increased in order to evaluate a total of 150 pigs. In order to record the resource-based indicator water supply, both the functionality and the flow rates of all nipple drinkers, bowls, and valves in pens that are selected for the animal welfare assessment according to the sampling should be checked.

For each indicator, the KTBL guide contains a description with information, exemplary photos and instructions for the collection and evaluation. The four indicators that have to be assessed for the whole fattening pig herd should be collected from the farm’s documentation: The result is a key figure for each of the four indicators: animal losses (%), treatment incidence antibiotics (sum of the number of treated animals times the number of treatment days for each type of antibiotic used within half a year), daily weight gains (g/d) and the proportion of animals affected per slaughter check (%). The indicators that are to be recorded for individual animals are differentiated according to different scores or categories (Table 2). For the analysis of the indicators tail length, lameness, faecal soiling, skin lesions, ear lesions, and tail lesions, the percentage of fattening pigs given the highest score of the indicator should be calculated in relation to the total number of animals examined. The relation between the number of fattening pigs identified as runts and the total number of animals examined, i.e., the percentage, is also used to evaluate this indicator. For the indicator evidence of ectoparasites, the decisive factor is whether or not there is a suspicion of infestation of the herd. For the resource-based indicator water supply, both the functionality and the flow rate of the sources are assessed. The result of recording this indicator is represented by the ratio between the number of water supply units with insufficient flow rates and the total number of units examined. The results are evaluated separately for all 13 animal welfare indicators. An aggregation of these values to form an overall welfare score as an overall result of animal welfare assessment is not included in the KTBL guide.

### 2.2. Structure of the Interview Guideline

The present study focuses on the collection of the individual assessments and opinions of farmers regarding the acceptance and feasibility of the KTBL guide “Animal Welfare Indicators: Practical Guide—Pigs” and its recommended indicators for fattening pigs. As no investigations into this had been previously undertaken, a qualitative research approach was chosen by conducting guided semi-structured interviews. On the one hand, the interview guideline provides a framework for carrying out the interviews and ensures that all necessary information is requested. On the other hand, the interview guideline guarantees a basic comparability between all conducted interviews. In total, the interview comprises 28 questions, which consist of 16 closed and 12 open questions. Thematically, the interview can be divided into four main sections: In the first section, general farm data and the farmers’ understanding of animal welfare are asked. The second section is about the on-farm self-assessment legally required by the Animal Welfare Act. In the third and most comprehensive section, questions about the farmer’s assessment of the KTBL guide are asked. Those questions concern both the KTBL guide as a broad method for animal welfare assessment, for example, its structure and comprehensibility, but also the specific contents, for example, the indicators and proposed procedures. The fourth and last section of the interview contains questions on approaches and ideas to improve the KTBL guide.

### 2.3. Interview Partners

In the context of the present study, all farmers with fattening pig husbandry in Germany belonged to the group of potential interview partners. In order to be able to represent a broad spectrum of opinions and assessments of the farmers, no further requirements were defined, e.g., about absolute herd sizes or the housing system. In total, interviews were carried out with 40 fattening pig farmers from the German States of Lower Saxony, North Rhine-Westphalia, Rhineland-Palatinate, and Saarland. All interviewed farmers operate conventional fattening pig husbandry. The fattening pigs were kept in insulated barns on 36 farms, three farms have outdoor climate housing and on one farm, the fattening pigs are kept in both outdoor climate pens and insulated pens. The farms had herds of between 200 and 6000 fattening pigs (mean 1780 ± standard deviation 1446). According to the group sizes, the farms could be subdivided: 18 farmers kept the fattening pigs in small groups, here defined as <20 pigs per pen. In total, 17 farmers had large groups of 20 to 60 fattening pigs per pen and five farmers run mega groups of more than 60 fattening pigs per pen.

### 2.4. Conduction of the Interviews

The KTBL guide “Animal Welfare Indicators: Practical Guide—Pigs” was sent to the 40 farmers per Email before the interviews were conducted and they were asked to read it through. The interviews were then undertaken face-to-face on the interviewees’ farms by three different people between June 2017 and May 2018. The interviews were conducted using the following procedure: After the interviewer had introduced him- or herself and had given short information about the study, the questions of sections one and two were discussed. Then, the farmer applied the KTBL guide and assessed the welfare of the fattening pigs on his farm in accordance with it. The interviewer supported her or him in this and answered his questions. This approach ensures that the farmers were in fact able to evaluate the feasibility of the KTBL guide and the indicators as well as the recommendations given. This guarantees that the famers’ assessments, opinions and evaluations that were recorded are of high quality. Afterwards, the remaining questions from sections three and four were asked. During the interviews the farmers’ answers were noted by hand. With the interviewee’s consent, the interviews were also recorded digitally to prevent any loss of information.

### 2.5. Evaluation of the Interviews

After the interviews were conducted, the data were first prepared and processed. For this purpose, the digital recordings of the interviews were listened to and the information was transcribed uniformly together with the handwritten notes. The answers to the closed questions were collected in tabular form in Microsoft Excel 2010 using a coding system. For evaluation, different procedures were used for closed and open questions. While the closed questions were evaluated descriptively, a qualitative content analysis according to Mayring was used to evaluate the open questions [12,13]. Therefore, the transcribed interviews were shortened and summarized in a first step without loss of content. In a second step, a system of answer categories was developed, which allowed a clear classification of individual passages of text. For this purpose, individual standard answers (i.e., quotations from the interviews) for each of the different categories were identified. These answers helped by clearly identifying which responses belonged to a specific category. In the third step, the individual passages of text from the farmers’ answers were assigned to the categories formed. This process was repeated several times, adapting the answer categories until all the information contained in the farmers’ statements could be clearly structured. Since a few questions remained unanswered by some of the farmers interviewed, the number of responses, i.e., the sample size, is indicated in the following presentation of the most important results of this study.

## 3. Results and Discussion

### 3.1. Acceptance of the KTBL Welfare Indicators

**Figure 1:**
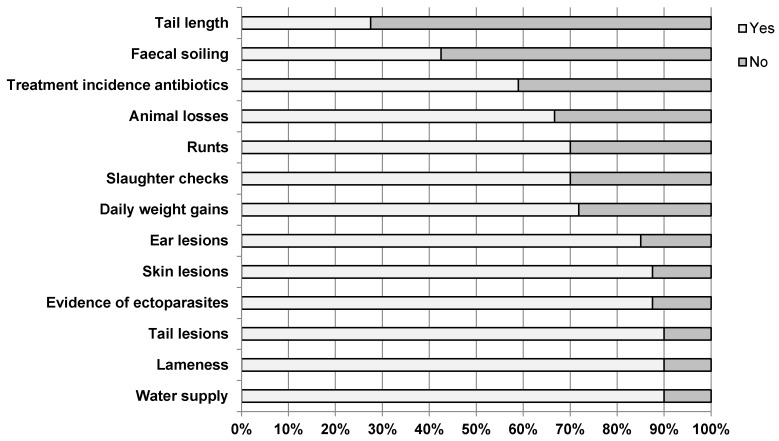
Farmers’ response to the question whether the 13 indicators can be considered as indicative with respect to fattening pig welfare or not (n = 40 responses, except for the indicators animal losses, treatment incidence antibiotics and daily weight gains n = 39 responses).

The KTBL guide proposes 13 indicators for the animal welfare assessment of fattening pigs. The interviewed fattening pig farmers were asked about their acceptance, i.e., whether or not they consider the individual indicator to be valid for the welfare assessment of fattening pigs. The result is shown in Figure 1 (*n* = 40 responses per indicator, except for the indicators animal losses, treatment incidence antibiotics and daily weight gains n = 39 responses). It shows that the farmers accept the proposed KTBL indicators to a varying extent for the animal welfare assessment of fattening pigs. The indicators water supply, lameness and tail lesions received the highest acceptance. All three indicators are considered indicative of the welfare of fattening pigs by 90% of the interviewed farmers. The acceptance of the indicators evidence of ectoparasites, skin lesions and ear lesions is only slightly lower, with ≥85% of the farmers seeing a correlation between the indicator and the welfare of fattening pigs. The indicators daily weight gains, slaughter checks, runts and animal losses are also considered valid for determining the welfare of fattening pigs by more than two-thirds of the farmers. More than half of the farmers (59%) also accepted the indicator treatment incidence antibiotics. In contrast, the majority of farmers reject the indicators faecal soiling and tail length. Only 42.5% of the interviewed farmers can identify a correlation between the faecal soiling of a fattening pig and its welfare. With regard to the indicator tail length, only 27.5% of the farmers state that the indicator is indicative for the well-being of fattening pigs.

The farmers were also asked to name the KTBL indicators which they consider to be the most or least meaningful with respect to the welfare of fattening pigs (n = 40 responses, multiple answers possible). In line with the answer to the previous question, the indicator faecal soiling was named 13 times as the indicator with the lowest degree of validity and the indicator tail length 12 times. The indicators slaughter checks, ear lesions, tail lesions, water supply and antibiotic treatment index were not mentioned once in this respect. The farmers were divided with regard to the indicator with the highest degree of validity. Apart from the indicator tail length, each of the 13 KTBL welfare indicators was mentioned at least once. A common consensus is that 16 people named at least one of the three indicators tail lesions, ear lesions or skin lesions as the KTBL indicator with the highest significance. Injuries to fattening pigs therefore appear to have a significant influence on the animals‘ welfare according to the farmers taking part in this study.

The question arises what causes the reduced acceptance of the indicators tail length and faecal soiling. Regarding the indicator tail length, the KTBL has two intentions with the proposal of this indicator [8]. One is to check the compliance with the legal requirements for tail docking in Germany (i.e., that only in exceptional cases a maximum of one third of a pig’s tail is docked). The other is to determine whether individual pigs have suffered further losses of tail length during rearing or fattening due to tail biting. However, the distinction has no influence on the fact that the farmers have a reduced acceptance of the indicator tail length. This becomes clear in the analysis of the farmers’ reasons for not accepting this indicator. Regarding the intention to reflect the result of tail docking, the farmers argue that the indicator is unsuitable for the welfare assessment of fattening pigs as docking is done by the piglet producer, who decides by which length the tails are shortened. Accordingly, the fattening pig farmer has just a limited influence on this and only give feedback to the piglet producer. With regard to the KTBL intention to use the indicator in order to detect losses of the length during the fattening period due to tail biting, the respondents have a clear attitude: Pigs that become victims of cannibalistic behaviour of penmates must be immediately separated from the group at the first signs. According to the interviewed farmers, it is unacceptable that such animals remain in the group and are attacked so severely that a significant loss of tail length occurs. In conclusion, the farmers interviewed within this study see only limited use and little relevance of the indicator tail length with respect to welfare assessment of fattening pigs.

Regarding the reduced acceptance of the welfare indicator faecal soiling, it must be said that its validity has also been questioned by scientists. In general, validity means that a method under investigation actually measures the specific characteristic it is supposed to measure [14]. In welfare science, only an indicator which truly provides information about animal welfare can be considered as valid [15]. For example, within a review of the currently used methods in animal welfare assessment, Winckler [16] points out that there is a lack of investigations on the topic whether or not indicators, collected during spot assessments, truly reflect the overall animal welfare. Winckler specifically mentions indicators regarding the degree of faecal pollution of animals in this context [16]. The reasoning of the farmers interviewed in this study goes along with this opinion. The example that was often cited by the interviewed farmers was that fattening pigs, which are contaminated with faeces to a greater extent because it is the first warm day of summer, do not necessarily suffer from reduced welfare.

### 3.2. Feasibility of the KTBL Welfare Indicators

In the KTBL guide, five indicators are proposed for the welfare assessment of fattening pigs, which are not recorded on the animals in the barn, but are collected from the farm’s documentation or technical equipment. The use of such data, which are recorded by default by most farmers anyway, is compliant with the KTBL’s aim of achieving the most positive cost-benefit ratio possible from the application of the guide for the farmer. In order to check the extent to which this objective has been realised, the fattening pig farmers were asked whether they record the data needed for those five indicators by default. The answers to this question can be summarised as follows (n = 40 answers per indicator): The indicators water supply (90%), slaughter checks (92.5%), animal losses (95%) and treatment incidence antibiotics (100%) are recorded by ≥90% of the interviewed fattening pig farmers by default. The indicator daily weight gains is routinely calculated by 75% of the farmers as an operational key figure. The percentage of the farmers who not only record those indicators by default but also evaluate them regularly is on average per indicator 10 percentage points lower (±6.1). In conclusion, the aim of the KTBL, to propose as many indicators as possible, which are recorded and evaluated by the farmers anyway, in order to increase the practical feasibility of the KTBL guide, has been achieved to a great extent.

**Figure 2:**
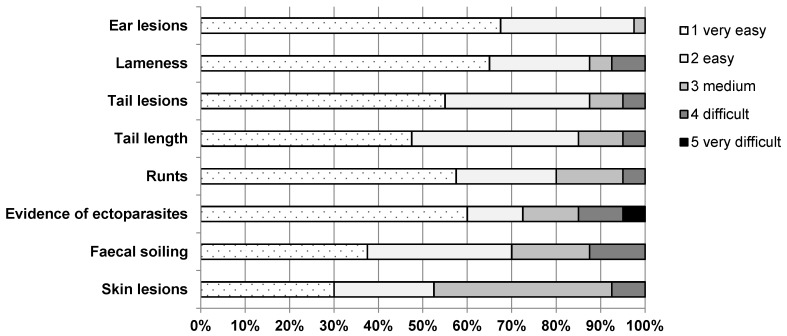
Assessment of the feasibility of the eight animal-based KTBL welfare indicators evaluated in individual animals on a five-point scale from very easy to very difficult according to the 40 fattening pig farmers interviewed in the present study (n = 40 ratings per indicator).

The feasibility of the KTBL indicators, which are recorded in the barn for individual pigs, was also judged by the 40 farmers interviewed. The farmers were asked to assess the feasibility of recording the eight animal-specific indicators on a five-point scale from very easy to very difficult. The result is shown in Figure 2 (*n* = 40 ratings per indicator). The animal-specific KTBL indicator which, in the opinion of the fattening pig farmers interviewed, can be recorded most easily, i.e., is most suitable for practical use, is the indicator ear lesions. In total, 97.5% of the farmers judged the assessment of this indicator as very easy or easy. The feasibility of the indicators runts, tail length, tail lesions and lameness is also quite high. The collection of these four indicators was rated as very easy or easy by 80% to 87.5% of the participants. In relation to this, the indicators evidence of ectoparasites, faecal soiling and skin lesions are less suitable for practical use. The proportion of farmers who rate the collection of these three indicators as very easy or easy is between 50% and 75%. The indicators evidence of ectoparasites and faecal soiling are particularly noticeable because more than 10% of the farmers described their collection as difficult or very difficult.

In the scientific literature, the feasibility of some of the eight animal-specific KTBL indicators is assessed in a similar way as in this study; for other indicators there are differences. Similar assessments can be found for the indicators ear lesions, tail lesions and skin lesions. With respect to the indicators ear lesions and tail lesions, the opinion of the 40 fattening pig farmers about the high feasibility of those two indicators is in line with the assessment of scientists. For example, Bracke [17] also judges the feasibility of these two indicators as relatively high. The collection of the indicator skin lesion was judged as medium or difficult by almost 50% of the interviewed farmers within this study. This is in accordance with Velarde [18], who describes that the feasibility of recording this indicator can be reduced depending on the situation. Examples of such situations are the collection in large groups of animals, in poor light conditions, in pens with high stocking densities, when there is a high degree of pollution of the animals, when the animals are resting and when the lesions are placed on the bottom of the pigs [18]. Regarding the indicator lameness, the farmers interviewed evaluated the feasibility differently than the scientists: Velarde [19], for example, considers the feasibility of recording this indicator as limited. The reason given is that for a valid evaluation of gait abnormalities during movement it is necessary to select the pigs from the pen, to separate them, to let them walk on a clean, dry and even floor and to carry out the evaluation under good light conditions [20]. That the feasibility of the indicator lameness was assessed as relatively high in this study is probably due to the fact that the KTBL guide does not make similar detailed instructions for the optimal recording of this indicator. In this respect, the KTBL guide just recommends that the collection should be carried out on non-slip flooring. The feasibility of the indicator faecal soiling was as well assessed contrarily by farmers interviewed in the study and scientists. According to Courboulay [21], this indicator can be collected from the inspection walkway of the pen with a high degree of feasibility even in large groups of pigs. However, a prerequisite for this is the recommendation that only one side of the animal needs to be checked for faecal soiling. Although this procedure is suggested identically in the KTBL guide, the feasibility of this indicator was rated as medium or difficult by 30% of the farmers within this study.

The interviewed fattening pig farmers justified the comparatively reduced feasibility of the indicators evidence of ectoparasites, faecal soiling and skin lesions by the following: some farmers stated that a trained eye, which has already seen an infestation, is required to recognise a suspicion of ectoparasites. This statement is in line with the reasoning of other respondents who admitted that there is a lack of experience and expertise in detecting a suspicion. Other fattening pig farmers pointed out that in order to see louse eggs the evaluator has to get very close to the animal and questioned the suitability of the KTBL recommendation that this should be done from a distance of one metre. To justify the comparatively low feasibility of the indicator skin lesions, the interviewed farmers referred to other challenges. On one hand, the farmers perceive the collection of this indicator as subjective, because there is a lack of clarity regarding the distinction of skin lesions. For example, questions have arisen as to when a scratch is considered a skin lesion, how to deal with dark, almost healed scabs or how to interpret skin reddening. On the other hand, as a justification for the reduced feasibility in practice, it was stated that the counting of the number of wounds to decide which score to give a pig was considered as being too time-consuming. Furthermore, the assessment of the length of a wound that is taken into account for the assignment of the three scores of the indicator from a distance was indicated as a challenge. As justification for difficulties in recording the indicator faecal soiling, most farmers mentioned the differentiation of the scores by percentage differences in the faecal contamination of the fattening pigs: many of the farmers interviewed said that it is easy to distinguish between clean and clearly contaminated pigs, but that the differentiation of score two of this indicator is a challenge and makes the collection of this indicator overall subjective.

**Figure 3:**
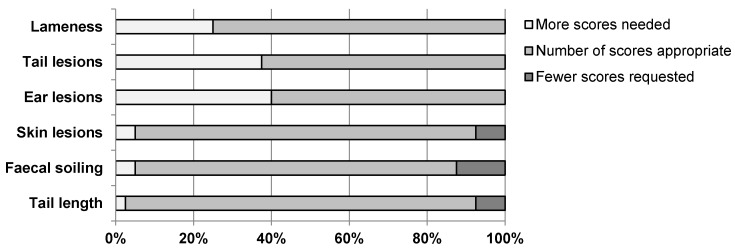
Judgement of 40 fattening pig farmers regarding the number of scores of six KTBL indicators suggested for welfare assessment of fattening pigs (n = 40 judgements per indicator).

Based on the arguments given by the farmers with respect to the assessment of the feasibility, questions about the effect of the number of scores within the indicators arise. It is remarkable that for the two indicators faecal soiling and skin lesions, whose collection was described as very easy or easy from a small number of farmers, not only two scores are distinguished, as in most other KTBL indicators, but three scores. During the interviews, the farmers were also asked to assess the number of scores within the indicators. The result is shown in Figure 3. First of all, it can be noted that for all indicators more than 60% of the farmers stated that the number of scores is appropriate and should not be changed. For the indicators lameness, tail lesions and ear lesions, in which two scores are distinguished, up to 40% of farmers identified a need for additional scores. The reason given for this is that with score one fattening pigs with significant abnormalities regarding these indicators are evaluated. The farmers are of the opinion that these pigs with clearly visible wounds and crusts on the ear or tail or clearly noticeable lameness should not be found in the regular herd at all, but should be housed in a hospital pen. Accordingly, farmers require a further score to detect low level tail or ear lesions and lameness. A proposal for this that needs to be examined regarding its effects on reliability, feasibility and validity of the indicators is: Score 0 of those three indicators could be defined as fattening pigs with normal gait respectively uninjured ears respectively uninjured tails without any abnormalities. Score 1 could be fattening pigs with a stiff gait, foreshortened stride, snake-like movements of the spine respectively ears with linear bite wounds or crusts or reddened ears respectively tails with linear bite wounds or crusts or reddened or slightly swollen tails. The idea would be that with the help of this score, victim animals could be identified early and separated from the group in order to prevent the occurrence of fattening pigs that have to be assessed with score 2, which is fattening pigs that do not put any weight on individual affected legs or are not able to walk at all respectively pigs with bleeding wounds or large scabs or intensely swollen and inflamed tails respectively ears. For the indicators skin lesions, faecal soiling and tail length, between 80% and 90% of the farmers interviewed rated the availability of three grades as appropriate. More scores for those three indicators were requested by ≤5% of the fattening pig farmers. In contrast, 7.5% to 12.5% of the farmers were of the opinion that the number of scores within these three indicators should be reduced. Thus, there are clearly fewer farmers who consider that the number of scores within the indicators faecal soiling and skin lesions should be reduced than respondents who describe the feasibility of collecting those two indicators as medium, difficult or very difficult.

### 3.3. Overall Feasibility of the KTBL Guide and Its Recommended Methods

In order to reduce the time and costs associated with the collection of the indicators for assessing fattening pig welfare, the KTBL guide proposes to take a random sample of the pigs of a herd into account. When asked whether this approach is permissible in terms of a valid assessment of the welfare level of an entire herd, 77.5% of the farmers agreed (n = 40 responses). The other 22.5% of the farmers have concerns in this respect and believe that in order to assess the welfare level of a herd accurately, all fattening pigs should be evaluated using the indicators. A study concerning the differences in the results between the assessment of the KTBL indicators in an entire herd of fattening pigs and in a random sample is already available [22]. In conclusion, the majority of the interviewed fattening pig farmers accept the KTBL proposal to consider a sample of pigs in welfare assessment. The sample size proposed in the KTBL guide (see Table 1) was evaluated by the farmers as follows (n = 40 answers): With 47.5%, almost half of the interviewed farmers felt that the recommended sample size was too high. In total, 40% of the fattening pig farmers are of the opinion that the recommended sample size is appropriate. In total, 12.5% of the farmers thought the sample size was too low and were willing to collect the indicators for a larger number of fattening pigs. These 12.5% interview partners have an average of 3560 ± 2533 fattening pig places on their farms, which is exactly twice as many fattening pigs than the average of all 40 farmers participating in this study. Among them are three farmers with 4600 to 6000 fattening pig places. The sample size of 150 pigs recommended in the KTBL guide means that for these three farms between 2.5% and 3.3% of the fattening pigs in their herds are taken into account to record the indicators faecal soiling, skin lesions, ear lesions, tail lesions, lameness and runts. However, two farmers with less than 1200 fattening places have also indicated that the sample size should be increased. For these two farmers, according to the current recommendations of the KTBL guide, 13% and 27.3% of the fattening pigs are taken into account when recording those animal-specific indicators. As a conclusion, further studies should investigate the effect of changes in the recommendations on sample size, for example, an increase or the consideration of a specific percentage of the fattening pigs in a herd for welfare assessment, on the feasibility and validity of the KTBL guide.

The fattening pig farmers also assessed the frequency suggested by the KTBL guide for collecting the indicators (see Table 1, *n* = 40 answers). In total, 60% of the interviewees were of the opinion that the proposed frequency of collection is appropriate and see no need for modification. That the indicators are to be collected too often was stated by 17.5% of the farmers, who would welcome a reduction of the frequency of collection. In total, 22.5% of the farmers interviewed thought that the indicators should be collected too seldom according to the KTBL guide and would be willing to record the indicators more often. In an additional question, the fattening pig farmers were asked to name specific indicators which should be more or less frequently evaluated (more than one answer possible, n = 40 farmers). More than 50% of the interviewees also answered this question by stating that no indicators should be collected more often or less frequently than recommended by the KTBL guide. The indicator tail lesions was named most often in connection with a wish for a higher frequency of collection (14 mentions). The indicators faecal soiling, lameness, skin lesions, ear lesions and runts were also mentioned in this context (5–10 mentions each). In contrast, the interviewees had no common opinion with respect to indicators that should be recorded less frequently than recommended in the KTBL guide. Only the indicator tail length was mentioned to a noteworthy extent (8 mentions). All other indicators were named only partially in this context (≤3 mentions each).

**Figure 4:**
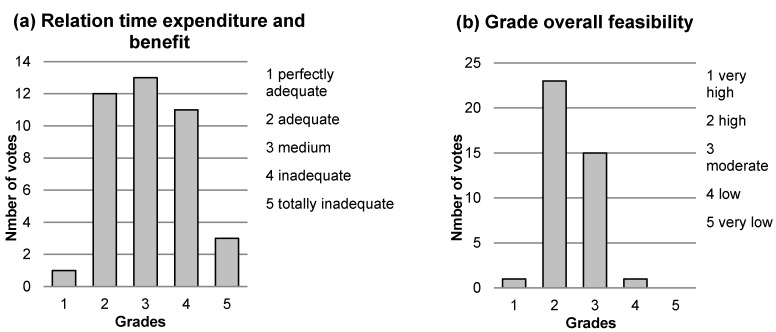
(**a**) Relation between time expenditure and benefit of applying the KTBL guide and (**b**) overall feasbility of the KTBL recommendations evaluated by 40 fattening pig farmers on five-point rating scales.

Within the scope of the interviews, the 40 fattening pig farmers were not only asked to evaluate the recommendations regarding the sampling and the frequency of the collection of the indicators, but also to judge on the feasibility of the KTBL guide as a complete set. In this context, the farmers were requested to assess the relation between the time expenditure needed to evaluate the welfare of the fattening pigs according to the KTBL guide and their benefits resulting from it on a five-point rating scale. In addition, the farmers were asked to rate the overall feasibility of the KTBL guide. The result of both questions is shown in Figure 4 (*n* = 40 responses each). The relation between time expenditure and benefit was judged as adequate by 12 farmers (grade 2), medium by 13 farmers (grade 3) and inadequate by 11 farmers (grade 4). The average rating was 3.1, which the farmers justified in the following way: the majority of the interviewed fattening pig farmers consider the time necessary to carry out the welfare assessment according to the KTBL guide as appropriate, but are dissatisfied with the resulting benefit. Many of the fattening pig farmers interviewed missed an overall result after performing the animal welfare assessment, i.e., an aggregation of the values for all individual indicators into a single value, like a key figure that can be classified and compared with others. After the indicators were collected, many farmers involved within this study asked questions about how to analyse and interpret the results and whether the level of animal welfare on their farm could be considered as being good or bad. This shows their lack of certainty about the informative value of the results of the welfare assessment according to the KTBL guide. The average overall assessment of the feasibility of the KTBL guide according to the 40 farmers was 2.4, with the majority of the farmers (57.5%) rating it high (grade 2) and a further 37.5% considering it to be moderate (grade 3). Despite this relatively good rating, the interviewees saw room for improvement in the KTBL guides’ recommendations and made concrete suggestions for modifications.

### 3.4. Potential for Improving the KTBL Guide According to the Interviewed Farmers

During the interviews, the farmers suggested various opportunities to improve the KTBL guide. Most of these suggestions have already been described above. However, four additional aspects are: (1) 77.5% of the interviewees expressed a wish for further tools to increase the efficiency and simplicity of animal welfare assessment according to the KTBL guide (n = 40 farmers). When asked which tool the farmers were thinking of (more than one response possible), the following two tools were mainly mentioned: Firstly, the farmers would like to have a standard copy template or scoring worksheet in which the recorded indicators can be easily noted while the evaluator is still in the barn (17 mentions). Secondly, the farmers desire a digital possibility for data entry, for example, an app that also automatically takes over the analysis of the data (17 mentions). (2) With regard to the evaluation of the result of the animal welfare assessment, farmers demand a standardised procedure to aggregate an overall result using all recorded indicators in order to increase the benefit from the application of the KTBL script. Such an overall result must clearly show the current status quo of animal welfare on the farm and also allow comparison with previous welfare assessments on the same farm or benchmarking with other farms. (3) Further suggestions for improvement can be derived regarding the set of indicators mentioned in the KTBL guide from the question if indicators should be excluded or included (*n* = 40 interviewed farmers). Additional indicators were suggested by 70% of the farmers. Most frequently named were parameters of the housing climate (a total of 12 mentions), such as air quality, concentration of harmful gases and temperature. The farmers stated that in their point of view these parameters have a huge influence on the welfare of the fattening pigs. Each of the following four indicators was mentioned three times: coughing and respiratory tract diseases, ocular and nasal discharge, umbilical and testicular hernias, and abnormalities affecting the limbs or joints, for example, bursitis. Only one mention each was made for the transportation time, the human–animal relationship and an indicator that captures the possibility of natural behaviours such as rooting and scratching. It is not surprising that the animal owners with several mentions suggested mainly resource-based indicators. Many studies report that farmers define animal welfare primarily in terms of good husbandry conditions [23]. When asked whether individual indicators should be omitted from the KTBL guide, only 32.5% of the farmers agreed. To a remarkable extent, only the indicator tail length was proposed for being omitted (6 mentions). (4) During the interviews, farmers repeatedly pointed out that the recommendations of the KTBL guide are very much focused on farms with conventional fattening pig husbandry in pens for up to 30 animals. The question arises whether the KTBL guide can be implemented easily on farms with straw bedding, organic farming and with large group sizes, for example, mega groups. As none of the interviewees is practising organic fattening pig farming and only four farmers with outdoor climate housing took part in the interviews, this question cannot be answered within the framework of the present study. In order to investigate this, further surveys should be carried out for specific target groups. The question of the feasibility of implementing the KTBL guide in mega groups cannot be answered finally either, because only five of the interview participants kept fattening pigs in mega groups with more than 60 animals. The main objection raised by these five farmers was that the KTBL guide does not contain recommendations for random sampling on farms with mega groups. For the sake of completeness, thoughts should be made about amending the KTBL recommendations for these types of fattening pig farms.

## 4. Conclusions

This study was carried out on 40 farms with fattening pigs husbandry. The participating farmers first used the KTBL publication “Animal Welfare Indicators: Practical Guide—Pigs” to assess the welfare of their fattening pigs and were then requested to evaluate the guide in semi-structured interviews. On the one hand, this qualitative study design ensures a high quality of the farmers’ statements and assessments. On the other hand, having interviewed 40 farmers out of the 18,900 to 19,700 fattening pig farms officially registered in Germany at the time of the data collection in 2017 and 2018 [24] means that the study has no claim to representativity. Thus, the results should be verified in further investigations with quantitative study designs and larger samples of the fattening pig farmers. The main results of this study are: The indicators proposed in the KTBL guide for the welfare assessment of fattening pigs are almost all accepted by a majority of the farmers interviewed. Exceptions are the indicators faecal soiling and tail length, whose significance for the welfare of fattening pigs is questioned by the farmers. The authors of the guide should critically consider whether to continue to recommend these two indicators. The feasibility of most of the indicators was assessed as good or very good, which means there is no need for action to change the KTBL guide. Consideration should be given to a change in the recommendations on sample size and the number of scores within the indicators lameness, ear lesions and tail lesions indicators. According to the farmers interviewed, there is a great need for action to provide tools for the efficient use of the KTBL guide. The farmers ask for a simple way to record the data when collecting the indicators, which also provides an analysis automatically. This analysis should allow farmers to determine the level of animal welfare on the farm and to compare the results over time or with other farms. In summary, a need for revision and further development of the KTBL guide was identified based on the results of this study. Thereby, the results of this study should be taken into account and the applicability of the possibilities suggested by the farmers to improve the guide should be examined.

## Figures and Tables

**Table 1:** Scheme for welfare assessment of fattening pigs contained in the KTBL guide. The table shows which indicators should be collected for which animals, when and how often (according to [8]).

Indicator	Time and Frequency of Collection and Analysis	The Animals to Be Assessed
Tail length	Collection with every new input of pigs and evaluation every six months	All newly housed fattening pigs
Animal losses Treatment incidence antibiotics ^1^ Daily weight gains Slaughter checks	Continuous collection and half-yearly analyses	All fattening pigs
Faecal soiling Skin lesions ^2^ Ear lesions Tail lesions Lameness Runts Evidence of ectoparasites Water supply	Collection and analysis semi-annually in the middle of the summer and winter half-year	Herds < 150 animals, all fattening pigs Herds >150 animals, random sample of the fattening pigs

^1^ to be evaluated on a quarterly basis if relevant. ^2^ at the earliest, collection one week after setting up new groups.

**Table 2:** Description of the scores or categories of the KTBL indicators which should be recorded for individual fattening pigs (according to [8]).

Indicators	Score or Category	Description
Tail length	0	Original length of the tail
	1	Remaining tail ≥2/3 of the original length
	2	Remaining tail <2/3 of the original length
Tail lesions	0	No observable wounds/scabs/swellings
	1	Clearly visible wounds/scabs/swellings
Faecal soiling	0	<10% of the body surface is soiled with faeces
	1	10 to 30% of the body surface is soiled with faeces
	2	>30% of the body surface is soiled with faeces
Skin lesions	0	<4 linear lesions with a length ≥5 cm and no lesions with a diameter ≥2.5 cm
	1	4–15 linear lesions with a length ≥5 cm and no lesions with a diameter ≥2.5 cm
	2	>15 linear lesions with a length ≥5 cm or at least one lesion with a diameter ≥2.5 cm
Ear lesions	0	No lesions or only some scratches on the external ear lobe but no larger wounds or scabs
	1	Clearly visible haemorrhagic wounds and scabs
Lameness	0	No or only a slight degree of lameness (stiff gait, foreshortened stride, snake-like movements of the spine)
	1	Obvious lameness (slight to severe reduction in weight-bearing) or ‘downer’ pig
Runts	No	Pig is clinically unremarkable
	Yes	Pig shows at least two of the following four characteristics: much smaller than the other animals in its group, protruding vertebrae, sunken flanks, long bristles
Evidence of	No suspicion	No sign of ectoparasites on any of the pigs
ectoparasites	Suspicion	There are pigs in the herd with lice, lice eggs, the initial signs of mange or full-scale mange
